# Unstructured spare time and crime: toward an integrative model

**DOI:** 10.1186/s40163-026-00276-y

**Published:** 2026-03-14

**Authors:** David Buil-Gil, Ken Pease

**Affiliations:** 1 Department of Criminology, The University of Manchester, Manchester, UK; 2 Department of Security and Crime Science, University College London, London, UK

**Keywords:** Leisure, Delinquency, Offending, Theoretical criminology, Environmental criminology

## Abstract

Criminological theorizing over the past half century has shown little convergence or integration. Three strands of criminological theory can be identified: dispositional approaches (emphasizing self-control, social learning, biological, and morality theories), ecological theories (emphasizing the crime consequences of dysfunctional communities), and opportunity theories (focusing primarily on places and artifacts that enable or facilitate crime). The discipline’s progress has not resulted in a convergence of theoretical propositions. This article offers a potential route toward reconciling these approaches, provisionally termed the Unstructured Spare Time model of crime. It begins with an overview of relevant criminological theories and highlights enduring tensions between individual- and opportunity-based approaches. It then reviews previous integrative efforts, noting their contributions and limitations. The Unstructured Spare Time model is introduced as a conceptual bridge among these traditions. The model posits that unstructured spare time, at the level of individuals, geographic areas, and time periods, is shaped by personal factors, broader social changes, and the spatial organization of cities and towns. This unstructured time, in turn, influences both individual readiness for crime and the availability of crime opportunities. The model advances a dynamic view of how time-use patterns mediate the relationship between personal traits, community conditions, structural factors, and exposure to and engagement in crime. Its central contribution lies in focusing explanation and, by extension, prevention and intervention strategies on a single, observable factor: unstructured spare time. The article summarizes empirical support from recent studies and concludes by outlining directions for future research and refinement of the model.

## Introduction

Theories of crime have, over decades, evolved into separate theoretical and empirical traditions, resulting in fragmented understandings of offending, victimization, and crime events. Optimal crime-control policy depends on integrating these strands. Dispositional approaches focus on variation between individuals in their propensity to commit crime, attributing this to biological (e.g., genetic, epigenetic, neurophysiological), psychological (e.g., mindfulness, impulsivity, self-control), or social-psychological (e.g., attachment to others, learned behaviors) characteristics. By contrast, situational approaches seek to explain how opportunities present in the immediate environment facilitate crime (Felson & Clarke, 1998; Mayhew et al., 1976). Ecological theories attend to how the social structures of local communities—such as social disorganization or neighborhood disadvantage—create conditions conducive to crime. Put crudely, dispositional theory seeks to change offenders, situational theory seeks to change places and objects, and ecological theory seeks to change the social dynamics of localities.

Each of the approaches outlined above has generated an extensive and valuable body of literature, but they offer only fragmented theoretical understandings of crime, with varying implications for crime policy and practice. Efforts to consolidate the theoretical landscape are long overdue. Integrative models matter not only for synthesizing evidence across frameworks but, crucially, for enabling evidence-based interventions grounded in agreed-upon criminological principles. If dispositional theories emphasize weak social bonds, low self-control, association with deviant peers, and poor moral reasoning; ecological theories focus on social cohesion and signs of disorder in the community; and situational approaches highlight offenders’ choices and the spatial convergence of targets and offenders—then how should we explain crime, and how should prevention proceed? Should we consider all these mechanisms simultaneously, or tailor our approach to the specific problem at hand? Navigating the pathways from theory to explanation, and from theory to practice, becomes—at best—an exercise in advanced theoretical literacy and, at worst, an insurmountable challenge for practitioners with limited access to academic knowledge.

Two main approaches to theoretical integration have been identified. Horizontal (or additive) integration involves combining mechanisms from multiple theories without reducing their complexity, while vertical (or reductionist/unificatory) integration seeks to reduce theories to their core principles, highlighting common underlying mechanisms.^1^ Horizontal integration seeks comprehensiveness by embracing complexity, whereas vertical integration aims for parsimony by distilling multiple theories into a unified set of core mechanisms. Both horizontal and vertical integration can occur within the same analytical level (e.g., combining two dispositional theories) or across multiple levels (e.g., linking individual traits with social environments). Horizontal, multi-level integration is common in criminology, as seen in models such as Elliott et al.’s (1979) Integrated Model of Delinquency, Thornberry’s (1987) Interactional Theory, and, more recently, Farrington’s (2005) Integrated Cognitive Antisocial Potential Theory. These integrative frameworks have played an essential role in consolidating knowledge about the causes and prevention of crime.

However, vertical integration poses a greater challenge and is arguably even more important. Unificatory efforts require demonstrating that multiple distinct factors, often rooted in entrenched theoretical traditions, can be meaningfully reduced to a small set of underlying mechanisms. This process demands rigorous conceptual and mechanistic synthesis, empirical evidence of causality or mediation, and often faces resistance from entrenched disciplinary traditions. Notable examples of vertical integration in the behavioral sciences include the Health Action Process Approach, which integrates psychological determinants of health behavior through the mediating role of ‘intention’ (Schwarzer, 1992); and psychotherapy’s common factors model, which posits the ‘therapeutic alliance’ as the central mechanism linking diverse therapeutic techniques to successful outcomes (Frank et al., 2025).

In this article, we propose the Unstructured Spare Time (UST) model as a vertical conceptual and theoretical bridge between competing perspectives in the study of crime. The model posits that unstructured spare time—at the level of individuals, geographic areas, and time periods—is shaped by personal factors, broader social changes, and the spatial organization of cities and towns. This unstructured time, in turn, influences both individual readiness for crime and the availability of criminal opportunities (Buil-Gil, 2025).^2^ The framework integrates principles from dispositional, ecological, and opportunity theories of crime around a single, observable factor: unstructured spare time, defined as time spent on activities for amusement, relaxation, or entertainment without predetermined agendas or goals (Abbott & Barber, 2007; Meeks & Mauldin, 1990; Osgood et al., 1996).

We move now to an overview of key criminological theories and the enduring tensions between individual, ecological, and opportunity-based explanations. The article then reviews previous integrative efforts, highlighting both their contributions and limitations, before describing the UST model in detail. Finally, it summarizes empirical support from recent studies and outlines directions for future research and model refinement.

## Theories of crime: conceptual and theoretical tensions

Dispositional, ecological, and situational theories of crime offer different explanations for why crime occurs, often resulting in conceptual tensions (see Table 1). The practical implications of these theoretical divisions for crime control policy are profound. Dispositional theories focus on individual-level factors, such as personality traits, self-control, or learned behaviors, inferring that crime stems from enduring characteristics or social-psychological processes within the person. In contrast, ecological theories emphasize the broader social environment, attributing crime to structural features such as weak social cohesion or community disorganization, which are thought to shape the social norms and opportunities available to individuals (Sampson et al., 1997; Shaw & McKay, 1942). Situational theories highlight the immediate circumstances that make crime possible or easy, such as the convergence of offenders, suitable targets and lack of guardians in a specific time and place (Cohen & Felson, 1979).

These divergent emphases create tensions not only over the causes of crime, but also over which factors are most important to address in prevention and intervention. Dispositional approaches prioritize changing people, often through education, treatment, or mentoring. Ecological theories suggest that effective prevention requires altering the broader social context, such as investing in community resources, strengthening neighborhood cohesion, or designing neighborhood policing initiatives. Meanwhile, situational approaches favor modifying environments or routines to limit opportunities for crime, through measures like target hardening, surveillance, or manipulating the physical space (Felson & Clarke, 1998; Mayhew et al., 1976). The tension arises because resources and policies are often directed based on which theoretical lens is prioritized, potentially leading to fragmented or incomplete responses. Integrating these perspectives remains a major challenge for creating comprehensive crime prevention strategies.

We argue that unstructured spare time may serve as a promising integrative concept, acting as a common pathway that links key mechanisms proposed by dispositional, ecological, and opportunity-based theories of crime. As described in Table 1, individual traits such as low self-control, weak social bonds, or certain personality profiles may predispose people to spend more time in unstructured—and potentially unsupervised or risky—activities (Hay & Forrest, 2008; Mahoney & Stattin, 2000; Pauwels & Svensson, 2011; Svensson & Oberwittler, 2010). Ecological factors such as social disorganization and collective efficacy can shape the availability and influence of unstructured spare time at the community level (Hoeben & Weerman, 2013; Maimon & Browning, 2010), while signs of disorder may attract individuals with greater amounts of unstructured time (Wilson & Kelling, 1982). From a situational perspective, unstructured routines may heighten the likelihood of convergence between offenders, suitable targets, and the absence of guardianship, directly shaping opportunities for crime (Cohen & Felson, 1979; Hindelang et al., 1978). Thus, unstructured spare time may offer a unifying framework that captures how individual predispositions, community context, and situational dynamics interact to influence criminal behavior. It is important to note, however, that while some empirical support exists for these propositions, they remain hypotheses and will need to be rigorously tested and validated in future research.

**Table 1:** Theories of crime and the hypothesized implications for unstructured spare time (only major theories are included)

Theory	Focus	Crime explanation (original theory)	Implications for unstructured spare time (hypothesized)
*Dispositional approaches*			
Personality trait theories (Eysenck, 1977)	Offender	Personality traits such as extraversion, neuroticism, and psychoticism make individuals more prone to offending.	Personality traits like extraversion, neuroticism, and psychoticism increase preference for stimulating, novel, and unsupervised unstructured activities.
Social bonds theory (Hirschi, 1969)	Offender	Crime is more likely when individuals lack strong attachments, commitments, involvement, and belief in conventional society.	Weak social bonds reduce both commitment to conventional goals and involvement in structured, prosocial activities.
Self-control theory (Gottfredson & Hirschi, 1990)	Offender	Low self-control leads to impulsive and risky behavior, including crime.	Low self-control leads to a preference for unstructured activities that offer short-term pleasure.
Social learning theories* (Akers, 1977)	Offender	Crime is learned through interaction with others and reinforcement.	Interaction with deviant peers is associated with a preference for unstructured, unsupervised activities.
Moral development theories (Kohlberg, 1984)	Offender	Poor moral reasoning limits the ability to judge right from wrong.	Poor moral reasoning increases risky use of unstructured spare time.
Strain theory* (Agnew, 1992)	Offender	Crime results from pressure due to blocked goals or negative experiences.	Those experiencing strain spend more time in unstructured activities as a way to cope with negative emotions.
*Ecological theories*			
Social disorganization theory (Shaw & McKay, 1942)	Communities	Crime is higher in areas with weak social structures and cohesion.	Disorganized areas provide more opportunities for unstructured unsupervised time for youth.
Collective efficacy theory (Sampson et el., 1997)	Communities	Strong social ties and informal control reduce crime.	High collective efficacy imposes greater structure and supervision over the spare time of locals and strangers.
Broken windows theory (Wilson & Kelling, 1982)	Communities	Signs of disorder invite further crime if left unchecked.	Signs of disorder attract individuals with more unstructured spare time.
*Opportunity and situational approaches*			
Routine activity theory (Cohen & Felson, 1979)	Crime event	Crime occurs when offenders, targets, and lack of guardians converge.	More unstructured time of offenders, targets, and guardians increases the chances of the convergence.
Lifestyle-exposure theory (Hindelang et al., 1978)	Victimization	Daily routines influence exposure to risky situations.	Unstructured daily routines raise exposure to risky situations.
Rational choice theory (Cornish & Clarke, 1986)	Crime event	Offenders weigh costs and benefits before acting.	Unstructured time provides more opportunities to weight costs and benefits and act.
Crime pattern theory (Brantingham & Brantingham, 1993)	Crime event	Crimes cluster in familiar places within offenders’ routines.	Unstructured time enables wider exploration of places and routine paths.

*Social learning and strain theories are often grouped separately from dispositional approaches, as they emphasize social interactions and environmental pressures rather than stable traits; however, both remain offender-centered and may be considered ‘dispositional’ if the category is defined broadly

### Past integrative efforts

Before describing the foundations of the proposed UST model, it is important to recognize and briefly review previous integrative models in crime studies, some of which incorporate principles from both dispositional and situational theories of crime. Most of these theoretical syntheses can be considered ‘horizontal’ integrations, as they combine mechanisms from multiple theoretical traditions without necessarily reducing their complexity.

Elliott et al.’s (1979) Integrated Model of Delinquency brings together social control, strain, and social learning theories, allowing for multiple pathways—such as weakened social bonds, experienced strain, or reinforcement by peer groups—through which delinquency can develop. Thornberry’s (1987) Interactional Theory integrates social control and social learning theories and introduces the concept of reciprocal effects, emphasizing that delinquency and social bonds influence each other over time. Braithwaite’s (1989) Reintegrative Shaming Theory synthesizes labeling theory and social bonds, arguing that the way society responds to crime—through stigmatization or reintegrative shaming—affects future offending. Farrington’s (2005) Integrated Cognitive Antisocial Potential Theory synthesizes cognitive, social, and developmental perspectives by focusing on risk factors that increase an individual’s potential for antisocial behavior across the life course. In the Spanish criminological literature, Redondo Illescas’s (2015) triple risk of crime model is gaining prominence as a framework that considers dispositional, social, and environmental risk factors to provide a comprehensive understanding of criminal risk. While these horizontal integrations broaden our understanding by combining elements from different theories, vertical integration is important because it seeks to unify diverse explanations under core underlying mechanisms, allowing for clearer theoretical and practical insights.

Wikström et al.’s (2012) Situational Action Theory exemplifies vertical integration by unifying individual propensities, environmental influences, and situational opportunities under a single explanatory mechanism: the perception-choice process. SAT posits that criminal behavior results from the interaction between a person’s crime propensity (shaped by factors such as moral values and self-control) and the criminogenic features of the settings they encounter, with action ultimately determined by how individuals perceive and choose to act in those situations. Central to SAT is the idea that individuals are not passively influenced by their environments. Instead, they actively interpret situational cues through a moral filter, assessing what actions are possible, acceptable, and desirable. In this way, SAT offers a unified framework for understanding how individual propensities and environmental contexts interact to produce criminal behavior. The proposed UST model complements this perspective by shifting analytical attention from the perception-choice process that governs cognitive responses in concrete situations to the ways in which individual, community, and structural factors shape patterns of unstructured spare time that determine readiness for crime and exposure to crime opportunities in the first place.

## Unstructured spare time model

The UST model proposes vertical integration of dispositional, ecological, and opportunity theories by linking individual traits identified in dispositional theories, community factors described by ecological theories, and situational antecedents theorized in opportunity perspectives to crime through a single, observable factor: unstructured spare time (Buil-Gil, 2025). Unstructured spare time refers specifically to time spent on activities for amusement, relaxation, or entertainment without predetermined agendas or goals. Examples include hanging out on the streets, attending parties, playing video games, or browsing social media, whether at home or elsewhere. Although spare time can provide important benefits for psychosocial development and well-being (Gilligan, 2000; Trainor et al., 2010; Winefield et al., 1992), research shows that when it is unstructured and extended in duration, it increases the risk of criminal involvement (Mahoney & Stattin, 2000; Beeck & Pauwels, 2010; Osgood, 2023). We thus distinguish unstructured spare time from structured spare time (e.g., sports, volunteering, family routines, extracurricular activities) and from committed time (e.g., sleeping, work, school), both of which are expected to offer protective benefits against crime.

Unstructured spare time extends beyond Osgood et al.’s (1996) concept of ‘unstructured socializing’ by encompassing all unscheduled or unplanned activities, not only those spent with peers. This expanded definition is supported on both theoretical and empirical grounds for four main reasons. First, although most research has emphasized unstructured socializing with peers (Deitzer et al., 2025; Engström, 2020; Ward & Forney, 2020), it is theoretically plausible that both unstructured time spent alone and with peers may increase exposure to criminogenic situations (Bernasco et al., 2013). Second, the growing amount of time individuals spend alone online generates extended periods of unstructured activity without peer interaction, during which young people may still encounter opportunities for risk-taking while being removed from protective routines. Third, time devoted to unstructured activities—whether solitary or social—replaces engagement in structured, supervised, and socially integrated activities that are associated with lower levels of offending (Badura et al., 2018; Bone et al., 2022; Mahoney et al., 2005). Finally, limiting the concept to peer-based socializing risks overemphasizing adolescence and neglecting important criminogenic dynamics that may operate in childhood (e.g., unsupervised time spent alone) and adulthood (e.g., unstructured time linked to substance use or gambling).

**Fig. 1:**
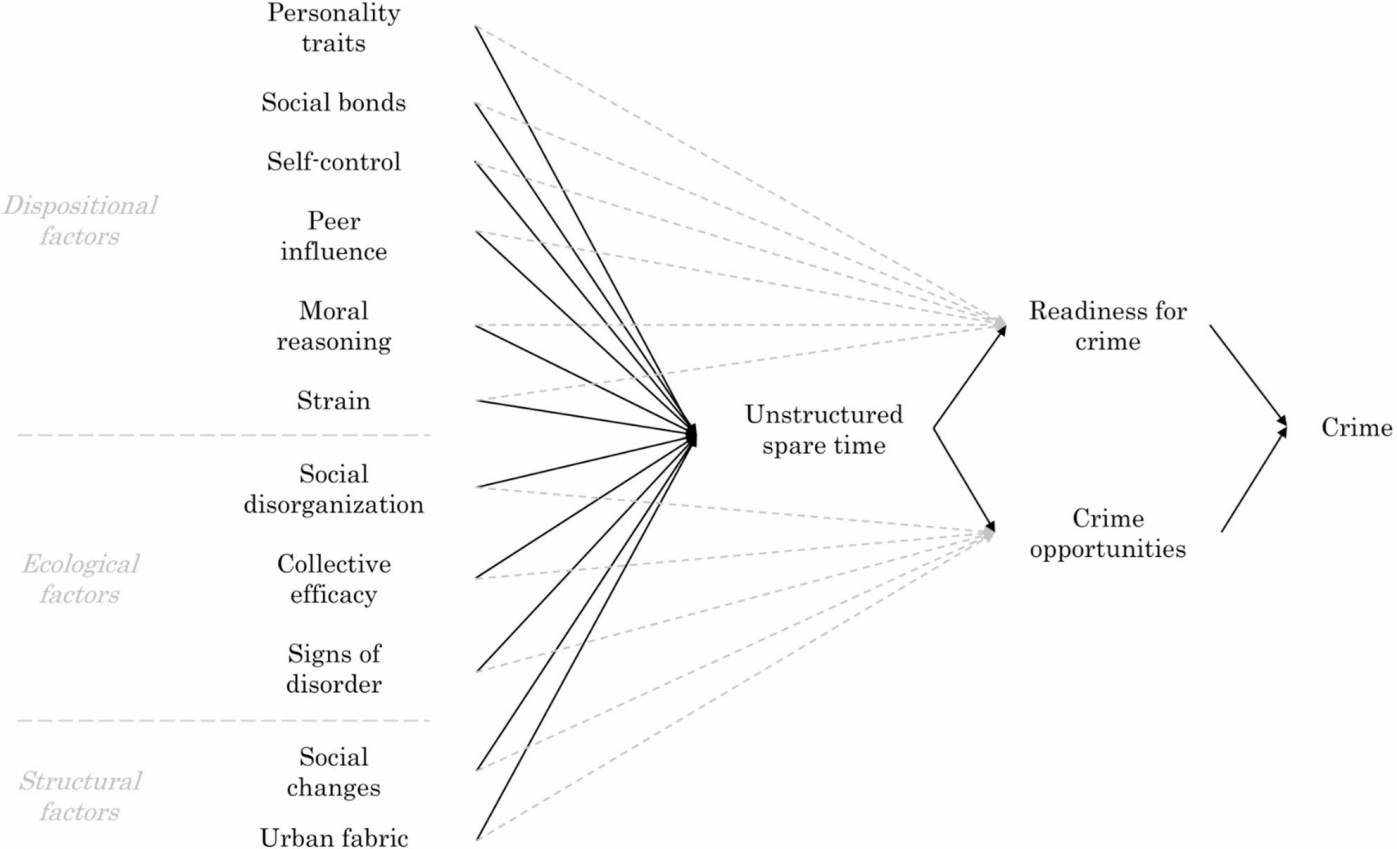
Theoretical diagram of Unstructured Spare Time (UST) model

The link between unstructured time and deviance or crime is not novel. The novelty of the UST model lies in integrating established findings about crime, derived from divergent theoretical traditions at different levels of analysis, to synthesize and unify crime explanations. Figure 1 presents the theoretical diagram of the model. The model proposes that dispositional, ecological, and structural factors shape the amount and characteristics of unstructured spare time for individuals, communities, and time periods, which in turn influences both individual readiness and opportunities for crime.

Specifically, drawing on dispositional theories—including personality trait theory (Eysenck, 1977), social bonds theory (Hirschi, 1969), self-control theory (Gottfredson & Hirschi, 1990), social learning theory (Akers, 1977), moral development theory (Kohlberg, 1984), and strain theory (Agnew, 1992)—we suggest that externalizing personality traits, weak social bonds, low self-control, deviant peer influence, poor moral reasoning, and experienced strain increase preferences for unstructured activities. Based on ecological theories such as social disorganization (Shaw & McKay, 1942), collective efficacy (Sampson et al., 1997), and broken windows (Wilson & Kelling, 1982), we emphasize that community conditions shape the availability, supervision, and social regulation of unstructured spare time. Finally, we propose that broader “social changes” (i.e., shifts in social and economic conditions) and variations in the “urban fabric” (i.e., the physical arrangement and organization of geographic areas within cities and towns) help explain temporal and spatial differences in unstructured leisure. This final set of structural factors is driven less by the integration of existing theoretical traditions than by well-established evidence on time use across historical periods and geographic contexts (e.g., Haleem et al., 2021; Wikström et al., 2012).

In turn, unstructured time facilitates crime not only by leaving more time available for deviant activity (Bernasco et al., 2013), but also—drawing on opportunity theories of crime—by reducing supervision and guardianship and increasing the presence of suitable targets (Cohen & Felson, 1979), heightening exposure to risky situations (Hindelang et al., 1978), enabling broader exploration of places where opportunities may arise (Brantingham & Brantingham, 1993), and providing more time to weigh costs and benefits before acting (Cornish & Clarke, 1986). Hoeben & Weerman (2016) further note that unstructured time with peers may increase individual tolerance for offending and substance use. We therefore use the generic term “readiness for crime” to encompass both predispositions and motivations to engage in crime, as well as other situational or contextual enablers that may trigger criminal behavior.

As indicated by the light gray arrows in the diagram, the UST model does not propose that the entire effect of these established criminological constructs on crime is mediated by unstructured spare time. There are, in fact, well-known mechanisms through which self-control, social learning, morality, and collective efficacy—to name just a few—can influence crime directly, independent of any pathways involving unstructured spare time. However, we argue that part of the effect of these antecedents of crime operates by modifying the unstructured spare time of individuals, communities, and time periods. This is precisely the unique strength of the model: centering part of the association between each antecedent and crime through unstructured spare time.

We also note that Fig. 1 omits all known and unknown links between dispositional factors (e.g., weak social bonds associated with differential associations with deviant peers), between ecological factors (e.g., collective efficacy being affected by social disorganization), between structural factors (e.g., social change affecting the layout of urban areas), as well as links between those levels (e.g., social learning facilitated by disorganized communities, signs of disorder affected by city layout, or individual strain affected by economic cycles). These links should be accounted for in empirical models wherever possible. Importantly, for crime to occur there must also be an interaction between an individual’s readiness for crime and the availability of crime opportunities; what Wikström et al. (2012) term the “person-environment interaction,” and, relatedly, what Cornish & Clarke (1986) describe with rationale choice theory. While the UST model focuses on the mediating role of unstructured time patterns that shape both readiness for crime and exposure to crime opportunities in the first place, it is essential to recognize that neither individual readiness nor opportunity alone is sufficient to explain crime. Rather, crime emerges from their interaction in specific contexts. Although this interaction is difficult to operationalize and measure empirically (Herrmann et al., 2025), it remains central to theoretical progress in understanding crime causation.

### Empirical support

Crime researchers have long recognized that unstructured time is a strong antecedent of crime. Hirschi (1969) argued that involvement in conventional activities limits opportunities for delinquency by occupying youths’ time with prosocial commitments. Hindelang et al. (1978) found that much of the difference in crime victimization is driven not only by how much spare time individuals have, but also by whether that time is structured and socially acceptable. Farrell & Pease (1993) noted that repeat victimization is strongly influenced by “chaotic lifestyles, occupations or leisure activities which make for continued vulnerability” (p. 7). Laub & Sampson (1993) observed that employment and marriage help reduce offending by decreasing unstructured routines: “those in stable employment and marital relations are typically subject to more structured routine activities and less free time than those in unstable roles” (p. 319). And Wikström et al.’s (2012) Situational Action Theory measures criminogenic exposure as “time spent in unstructured peer-oriented activities” (p. 147). Evidence linking unstructured spare time and crime is strong and cross-nationally consistent (Mahoney & Stattin, 2000; Mahoney et al., 2004; Pauwels & Svensson, 2011), including in low- and middle-income countries (Buil-Gil et al., 2025). In contrast, structured spare time—like education, sports, and family routines—fosters prosocial values and encourages long-term goals (Badura et al., 2018; Bone et al., 2022; Mahoney et al., 2005).

There is also emerging evidence linking dispositional factors with unstructured spare time, with some studies showing that the effect of these factors on crime operates through unstructured spare time. Hay & Forrest (2008) analyzed a sample of juveniles and found that the effect of self-control on delinquency partially depends on time spent with friends and away from supervision. Mahoney & Stattin (2000) found that participation in unstructured activities was associated with deviant peer relations and poor parent-child relations, with unstructured time being strongly linked to antisocial behavior. Archer et al. (2022) found that unstructured socializing mediates the effect of parental monitoring on youth delinquency (see also Osgood & Anderson, 2004). Beeck & Pauwels (2010) observed that certain sources of strain, but not all, lead to adolescent offending only by modifying unstructured routines. Svensson & Oberwittler (2010) analyzed samples of young adolescents in Sweden and Germany, concluding that the effect of having delinquent peers on offending is conditional on the amount of time spent in unstructured routines (see also Gerstner & Oberwittler, 2018; Hoeben & Weerman, 2016). And Chrysoulakis et al. (2022) found that adolescents with weaker morality are more likely to break rules during unstructured socializing than those with stronger morality.

Ecological and structural factors have also been found to influence crime by modifying unstructured routines at the community and societal levels. Wikström et al. (2012) found that community variables such as social disadvantage and land use strongly influence young people’s unstructured time, which in turn is closely associated with police-recorded crime in small areas. Maimon & Browning (2010) analyzed longitudinal survey data from Chicago and found that community social control and cohesion (collective efficacy) influence both unstructured time use and the association between unstructured socializing and violent behavior by providing informal monitoring. Hoeben & Weerman (2013) also found that the type of place influences the association between unstructured time and offending.

Multiple studies have shown that temporal changes in unstructured time help explain trends in crime (Baumer et al., 2021; Buil-Gil, 2025; Miró-Llinares, 2025; Oberwittler & Svensson, 2025; Osgood, 2023; Svensson & Oberwittler, 2021).

## Further work

The UST model proposes a vertically integrated framework that synthesizes dispositional, ecological, and opportunity theories by identifying a common pathway through which their mechanisms lead to crime: unstructured spare time. By tracing how diverse influences converge through time use, the UST model bridges theoretical divides and points toward concrete strategies for crime prevention.

In practice, the UST model suggests that prevention strategies should focus on the availability and quality of structured activities for individuals and communities. Interventions that redirect unstructured spare time—especially in public or unsupervised settings—toward supervised, meaningful activities may be particularly effective for high-risk youth, potentially reducing crime and associated social costs. Efforts can and should operate at multiple levels: parents and caregivers can be supported in fostering structured routines at home; communities can invest in accessible youth centers, sports, and arts programs; schools play a vital role through after-school initiatives and mentoring schemes, while exclusions from school increase risk (Cornish & Brennan, 2025); and local authorities can enhance access to safe public spaces and reliable transport. Importantly, such initiatives should not aim to curtail adolescent autonomy, but rather to expand the availability of safe, structured, and developmentally enriching options. The model also highlights the negative consequences of disinvestment in youth services, suggesting that cuts to organized leisure provision may contribute to rising levels of youth crime. Collectively, the model underscores that the structure of individuals’ spare time is both a salient risk factor for crime and a promising focus for prevention.

Despite growing evidence to support the UST model, much remains to be tested. While emerging studies substantiate some of the mechanisms linking dispositional, ecological, and structural factors with unstructured spare time, these propositions remain, in many respects, hypotheses that require robust empirical validation. Longitudinal data are essential for establishing causal pathways, though such data are often costly to collect; linked administrative data collected in educational and criminal justice settings offer a valuable resource for longitudinal analysis without the limitations and costs of traditional sampling (Buil-Gil & Pease, 2025). It is also important to advance theoretical understanding of the cognitive processes through which individual readiness for crime and crime opportunities interact in specific circumstances to produce crime (Cornish & Clarke, 1986; Wikström et al., 2012).

Future work should explore the spatial heterogeneity of unstructured spare time and its relationship with crime, recognizing that local contexts may modify these links. From a situational perspective, it is important to examine not only the unstructured time of offenders, but also that of victims and guardians, as their routines also shape crime opportunities. Moreover, one could argue that while the proposed model is readily applicable to adolescent street and youth crime, its relevance to other forms of offending—such as white-collar and corporate crime, terrorism, and state crime—has clearer boundaries. For us, this remains an open question. There is emerging evidence, for instance, that radicalization is more common among individuals disengaged from school and work, and thus with more limited access to structured time (Weenink, 2019). Similarly, Zeng (2021) shows that the information sharing necessary for insider dealing and related white-collar offences often takes place in low-structure leisure settings such as pubs and informal social gatherings. Future research should therefore reconsider how the UST model might apply to forms of crime beyond street and youth offending, examining whether unstructured spare time operates differently in organizational or professional contexts and how opportunity structures manifest in those settings. Finally, as technological advances and the expansion of digital environments continue to reshape how and where individuals spend their time, future research must address how these changes influence unstructured spare time and its links to both risk and protection.

## Data Availability

Not applicable.

## Footnotes

1 Distinctions between additive and unificatory forms of theoretical integration have long been discussed in the philosophy of science, particularly in relation to multi-level explanation and the organization of causal mechanisms (e.g., Bechtel & Richardson, 2000; Merton, 1968; Wimsatt, 2007). In the present article, we introduce the terms horizontal and vertical integration to clarify two contrasting strategies of theoretical synthesis in criminology: one that combines multiple mechanisms without reducing their complexity, and another that seeks to identify common underlying pathways across theoretical traditions. While the original authors of the criminological models reviewed here did not necessarily use this terminology, the distinction provides a useful heuristic for clarifying different forms of theoretical synthesis.

2 The present article builds on earlier work (Buil-Gil, 2025), which introduced the foundations of the UST model and provided initial empirical evidence of the association between unstructured spare time and offending using cross-national survey data collected in the United States. The current paper differs in both scope and purpose. Here, we develop and expand the UST model as an articulated theoretical framework, situating it explicitly within the broader criminological literature on dispositional, ecological, and opportunity theories of crime. Whereas earlier empirical work focused primarily on individual-level control, self-control, social learning, and opportunity theories, the present study substantially broadens the scope of integration. We further elaborate the model’s conceptual foundations, clarify its integrative logic, and discuss its implications for theory-building and crime prevention.

## References

[CR1] Abbott, B. D., & Barber, B. L. (2007). Not just idle time: Adolescents’ developmental experiences provided by structured and unstructured leisure activities. *The Educational and Developmental Psychologist*, *24*, 59–81. 10.1017/S0816512200029102

[CR2] Agnew, R. (1992). Foundation for a general strain theory of crime and delinquency. *Criminology*, *30*(1), 47–87. 10.1111/j.1745-9125.1992.tb01093.x

[CR3] Akers, R. L. (1977). *Deviant Behavior: A Social Learning Approach* (2nd ed.). Wadsworth.

[CR4] Archer, R., Jackson-Jefferson, M., Celebi, M., & Granger, T. (2022). Understanding the role of unstructured socializing with peers and peer delinquency as mediators in the relationship of parental monitoring and delinquency. *Journal of Crime and Justice*, *45*(5), 588–608. 10.1080/0735648X.2022.2038232

[CR5] Badura, P., Madarasova Geckova, A., Sigmundova, D., Sigmund, E., van Dijk, J. P., & Reijneveld, S. A. (2018). Can organized leisure-time activities buffer the negative outcomes of unstructured activities for adolescents’ health? *International Journal of Public Health*, *63*, 743–751. 10.1007/s00038-018-1125-3 29860658 10.1007/s00038-018-1125-3 PMC6015610

[CR6] Baumer, E. P., Cundiff, K., & Luo, L. (2021). The contemporary transformation of american youth: An analysis of change in the prevalence of delinquency, 1991–2015. *Criminology*, *59*(1), 109–136. 10.1111/1745-9125.12264 36776699 10.1111/1745-9125.12264 PMC9910102

[CR7] Bechtel, W., & Richardson, R. C. (2000). *Discovering Complexity: Decomposition and Localization as Strategies in Scientific Research*. MIT Press.

[CR8] Bernasco, W., Ruiter, S., Bruinsma, G. J., Pauwels, L. J., & Weerman, F. M. (2013). Situational causes of offending: A fixed-effects analysis of space–time budget data. *Criminology*, *51*(4), 895–926. 10.1111/1745-9125.12023

[CR9] Bone, J. K., Bu, F., Fluharty, M. E., Paul, E., Sonke, J. K., & Fancourt, D. (2022). Arts and cultural engagement, reportedly antisocial or criminalized behaviors, and potential mediators in two longitudinal cohorts of adolescents. *Journal of Youth and Adolescence*, *51*, 1463–1482. 10.1007/s10964-022-01591-8 35318575 10.1007/s10964-022-01591-8 PMC8940513

[CR10] Braithwaite, J. (1989). *Crime, Shame and Reintegration*. Cambridge University Press.

[CR11] Brantingham, P. J., & Brantingham, P. L. (1993). Environment, routine, and situation: toward a pattern theory of crime. *Advances in Criminological Theory*, *5*, 259–294.

[CR12] Buil-Gil, D. (2025). The structure of unstructured time and crime: A spare time model. *British Journal of Criminology*, *66*, 1–22. 10.1093/bjc/azaf035

[CR14] Buil-Gil, D., & Pease, K. (2025). *From School Absences to Crime Involvement*. Data Insight. ADR UK.

[CR13] Buil-Gil, D., Birkbeck, C., Enzmann, D., Arbach, K., R Bazon, M., Budimlić, M., & H Marshall, I. (2025). Unstructured spare time as an international predictor of adolescent crime. *CrimRxiv*. 10.21428/cb6ab371.e243b32e 10.1371/journal.pone.0349291 PMC13178864 42139201

[CR18] Chrysoulakis, A. P., Ivert, A. K., & Levander, M. T. (2022). From Structural time use to situational rule-breaking: Analysing adolescents’ time use and the person-setting interaction. *European Journal of Criminology*, *20*(6), 1804–1828. 10.1177/14773708221097657

[CR15] Cohen, L. E., & Felson, M. (1979). Social change and crime rate trends: A routine activity approach. *American Sociological Review*, *44*(4), 588–608. 10.2307/2094589

[CR17] Cornish, R., & Brennan, I. (2025). Exclusion from school and risk of serious violence: A target trial emulation study. *British Journal of Criminology*, *65*(6), 1221–1240. 10.1093/bjc/azaf015

[CR16] Cornish, D. B., & Clarke, R. V. (Eds.). (1986). *The Reasoning Criminal: Rational Choice Perspectives on Offending*. Springer.

[CR48] de Op Beeck, H., & Pauwels, L. (2010). Do unstructured routines modify the link between social-psychological strain and adolescent offending? *European Journal on Criminal Policy and Research*, *16*, 221–235. 10.1007/s10610-010-9127-6

[CR19] Deitzer, J. R., McGloin, J. M., & Hoeben, E. (2025). Specifying the temporal bounds of the situational peer effect. *Journal of Research in Crime and Delinquency*, *62*(5), 703–743. 10.1177/00224278251338774

[CR20] Elliott, D. S., Ageton, S. S., & Canter, R. J. (1979). An integrated theoretical perspective on delinquent behavior. *Journal of Research in Crime and Delinquency*, *16*(1), 3–27. 10.1177/002242787901600102

[CR21] Engström, A. (2020). Conceptualizing lifestyle and routine activities in the early 21st century: A systematic review of self-report measures in studies on direct-contact offenses in young populations. *Crime & Delinquency*, *67*(5), 737–782. 10.1177/0011128720937640

[CR22] Eysenck, H. J. (1977). *Crime and Personality* (3rd ed.). Routledge.

[CR23] Farrell, G., & Pease, K. (1993). *Once Bitten, Twice Bitten: Repeat Victimisation and Its Implications for Crime Prevention*. Home Office Police Department. Crime Prevention Unit Series Paper No. 46.

[CR24] Farrington, D. P. (2005). The integrated cognitive antisocial potential (ICAP) theory. In D. P. Farrington (Ed.), *Integrated Developmental and Life-course Theories of Offending* (pp. 73–92). Transaction.

[CR25] Felson, M., & Clarke, R. V. (1998). *Opportunity Makes the Thief: Practical Theory for Crime Prevention*. Research Development Statistics. Police Research Series Paper 98.

[CR26] Frank, J. D., Frank, J. B., & Wampold, B. E. (2025). *Persuasion and Healing: A Comparative Study of Psychotherapy* (updated ed.). John Hopkins University Press.

[CR27] Gerstner, D., & Oberwittler, D. (2018). Who’s hanging out and what’s happening? A look at the interplay between unstructured socializing, crime propensity and delinquent peers using social network data. *European Journal of Criminology*, *15*(1), 111–129. 10.1177/1477370817732194

[CR28] Gilligan, R. (2000). Adversity, resilience and young people: The protective value of positive school and spare time experiences. *Children & Society*, *14*(1), 37–47. 10.1111/j.1099-0860.2000.tb00149.x

[CR29] Gottfredson, M. R., & Hirschi, T. (1990). *A General Theory of Crime*. Stanford University Press.

[CR30] Haleem, M. S., Lee, D., Ellison, W., M., & Bannister, J. (2021). The exposed population, violent crime in public space and the night-time economy in Manchester, UK. *European Journal on Criminal Policy and Research*, *27*, 335–352. 10.1007/s10610-020-09452-5

[CR31] Hay, C., & Forrest, W. (2008). Self-control theory and the concept of opportunity: The case for a more systematic union. *Criminology*, *46*(4), 1039–1072. 10.1111/j.1745-9125.2008.00135.x

[CR32] Herrmann, C., Uhl, A., & Treiber, K. H. (2025). Peeking into the black box of offender decision-making: A novel approach to testing situational action theory’s perception choice process. *Deviant Behavior*. 10.1080/01639625.2025.2494144

[CR33] Hindelang, M. J., Gottfredson, M. R., & Garofalo, J. (1978). *Victims of Personal Crime: An Empirical Foundation for a Theory of Personal Victimization*. Ballinger.

[CR34] Hirschi, T. (1969). *Causes of Delinquency*. University of California Press.

[CR35] Hoeben, E., & Weerman, F. (2013). Situational conditions and adolescent offending: Does the impact of unstructured socializing depend on its location? *European Journal of Criminology*, *11*(4), 481–499. 10.1177/1477370813509346

[CR36] Hoeben, E. M., & Weerman, F. M. (2016). Why is involvement in unstructured socializing related to adolescent delinquency? *Criminology*, *54*(2), 242–281. 10.1111/1745-9125.12105

[CR37] Kohlberg, L. (1984). *Essays on Moral Development: Vol. II. The Psychology of Moral Development*. Harper & Row.

[CR38] Laub, J. H., & Sampson, R. J. (1993). Turning points in the life course: Why change matters to the study of crime. *Criminology*, *31*(3), 301–325. 10.1111/j.1745-9125.1993.tb01132.x

[CR39] Mahoney, J. L., & Stattin, H. (2000). Leisure activities and adolescent antisocial behavior: The role of structure and social context. *Journal of Adolescence*, *23*(2), 113–127. 10.1006/jado.2000.0302 10831137 10.1006/jado.2000.0302

[CR40] Mahoney, J. L., Stattin, H., & Lord, H. (2004). Unstructured youth recreation centre participation and antisocial behaviour development: Selection influences and the moderating role of antisocial peers. *International Journal of Behavioral Development*, *28*(6), 553–560. 10.1080/01650250444000270

[CR41] Mahoney, J. L., Larson, R. W., & Eccles, J. S. (2005). *Organized Activities as Contexts of Development: Extracurricular Activities, After-School and Community Programs*. Lawrence Erlbaum.

[CR42] Maimon, D., & Browning, C. R. (2010). Unstructured socializing, collective efficacy, and violent behavior among urban youth. *Criminology*, *48*, 443–474. 10.1111/j.1745-9125.2010.00192.x

[CR43] Mayhew, P., Clarke, R. V. G., Sturman, A., & Hough, J. M. (1976). *Crime as Opportunity*. Home Office Research Study No. 34. Her Majesty’s Stationery Office.

[CR44] Meeks, C. B., & Mauldin, T. (1990). Children’s time in structured and unstructured leisure activities. *Journal of Family and Economic Issues*, *11*, 257–281. 10.1007/BF00987003

[CR45] Merton, R. K. (1968). *Social Theory and Social Structure*. Simon and Schuster.

[CR46] Miró-Llinares, F. (2025). Crime opportunities, lockdowns, and online video games: The digital leisure hypothesis (and more on the impact of digitalization on crime trends). In M. F. Aebi, F. Miró-Llinares, & S. Caneppele (Eds.), *Understanding Crime Trends in a Hybrid Society* (pp. 77–100). Springer.

[CR47] Oberwittler, D., & Svensson, R. (2025). The international youth crime drop: Evidence and explanations. *Criminal and Justice*, *54*, 153–216. 10.1086/737409

[CR49] Osgood, D. W. (2023). Delinquency, unstructured socializing, and social change: The rise and fall of a teen culture of independence. *Criminology*, *61*(4), 681–704. 10.1111/1745-9125.12358

[CR50] Osgood, D. W., & Anderson, A. L. (2004). Unstructured socializing and rates of delinquency. *Criminology*, *42*(3), 519–550. 10.1111/j.1745-9125.2004.tb00528.x

[CR51] Osgood, D. W., Wilson, J. K., O’Malley, P. M., Bachman, J. G., & Johnston, L. D. (1996). Routine activities and individual deviant behavior. *American Sociological Review*, *61*, 635–655. 10.2307/2096397

[CR52] Pauwels, L. J. R., & Svensson, R. (2011). Exploring the relationship between offending and victimization: What is the role of risky lifestyles and low self-control? A test in two urban samples. *European Journal on Criminal Policy and Research*, *17*, 163–177. 10.1007/s10610-011-9150-2

[CR53] Redondo Illescas, S. (2015). *El Origen de los Delitos: Introducción al Estudio y Explicación de la Criminalidad*. Tirant lo Blach.

[CR54] Sampson, R. J., Raudenbush, S. W., & Earls, F. (1997). Neighborhoods and violent crime: A multilevel study of collective efficacy. *Science*, *277*(5328), 918–924. 10.1126/science.277.5328.918 9252316 10.1126/science.277.5328.918

[CR55] Schwarzer, R. (1992). Self-efficacy in the adoption and maintenance of health behaviors: Theoretical approaches and a new model. In R. Schwarzer (Ed.), *Self-Efficacy: Thought Control of Action* (pp. 217–243). Hemisphere.

[CR56] Shaw, C. R., & McKay, H. D. (1942). *Juvenile Delinquency and Urban Areas*. University of Chicago Press.

[CR57] Svensson, R., & Oberwittler, D. (2010). It’s not the time they spend, it’s what they do: The interaction between delinquent friends and unstructured routine activity on delinquency: Findings from Two Countries. *Journal of Criminal Justice*, *38*(5), 1006–1014. 10.1016/j.jcrimjus.2010.07.002

[CR58] Svensson, R., & Oberwittler, D. (2021). Changing routine activities and the decline of youth crime: A repeated cross-sectional analysis of self-reported delinquency in Sweden, 1999–2017. *Criminology*, *59*(2), 351–386. 10.1111/1745-9125.12273

[CR59] Thornberry, T. P. (1987). Toward an interactional theory of delinquency. *Criminology*, *25*(4), 863–892. 10.1111/j.1745-9125.1987.tb00823.x

[CR60] Trainor, S., Delfabbro, P., Anderson, S., & Winefield, A. (2010). Leisure activities and adolescent psychological well-being. *Journal of Adolescence*, *33*(1), 173–186. 10.1016/j.adolescence.2009.03.013 19406463 10.1016/j.adolescence.2009.03.013

[CR67] Ward, J. T., & Forney, M. (2020). Unpacking within-and between-personeffects of unstructured socializing and differential association on solo-and co-offending. Journal of Criminal Justice, 70, 101720. 10.1016/j.jcrimjus.2020.101720

[CR61] Weenink, A. W. (2019). Adversity, criminality, and mental health problems in Jihadis in Dutch police files. *Perspectives on Terrorism*, *13*(5), 130–142.

[CR62] Wikström, P. H., Oberwittler, D., Treiber, K., & Hardie, B. (2012). *Breaking Rules: The Social and Situational Dynamics of Young People’s Urban Crime*. Oxford University Press.

[CR63] Wilson, J. Q., & Kelling, G. L. (1982). Broken windows. *Atlantic Monthly*, *249*(3), 29–38.

[CR64] Wimsatt, W. C. (2007). *Re-Engineering Philosophy for Limited Beings*. Harvard University Press.

[CR65] Winefield, A. H., Tiggemann, M., & Winefield, H. R. (1992). Spare time use and psychological well-being in employed and unemployed young people. *Journal of Occupational and Organizational Psychology*, *65*(4), 307–313. 10.1111/j.2044-8325.1992.tb00507.x

[CR66] Zeng, Y. (2021). *Organising Insider Dealing in Financial Markets: Scripts and Networks.* PhD thesis, University of Manchester.

